# Supplementary material for: Novel Driver Strength Index highlights important cancer genes in TCGA PanCanAtlas patients
Source: PeerJ. 2022 Aug 11;10:e13860. doi: 10.7717/peerj.13860 (PMC9375969; doi:10.7717/peerj.13860)
Supplement: Supplemental Information 2 [file peerj-10-13860-s002.zip › COHORTS/genes plots/age/2021_8_16_14_9_distribution_cohorts_females_45-49.pdf]

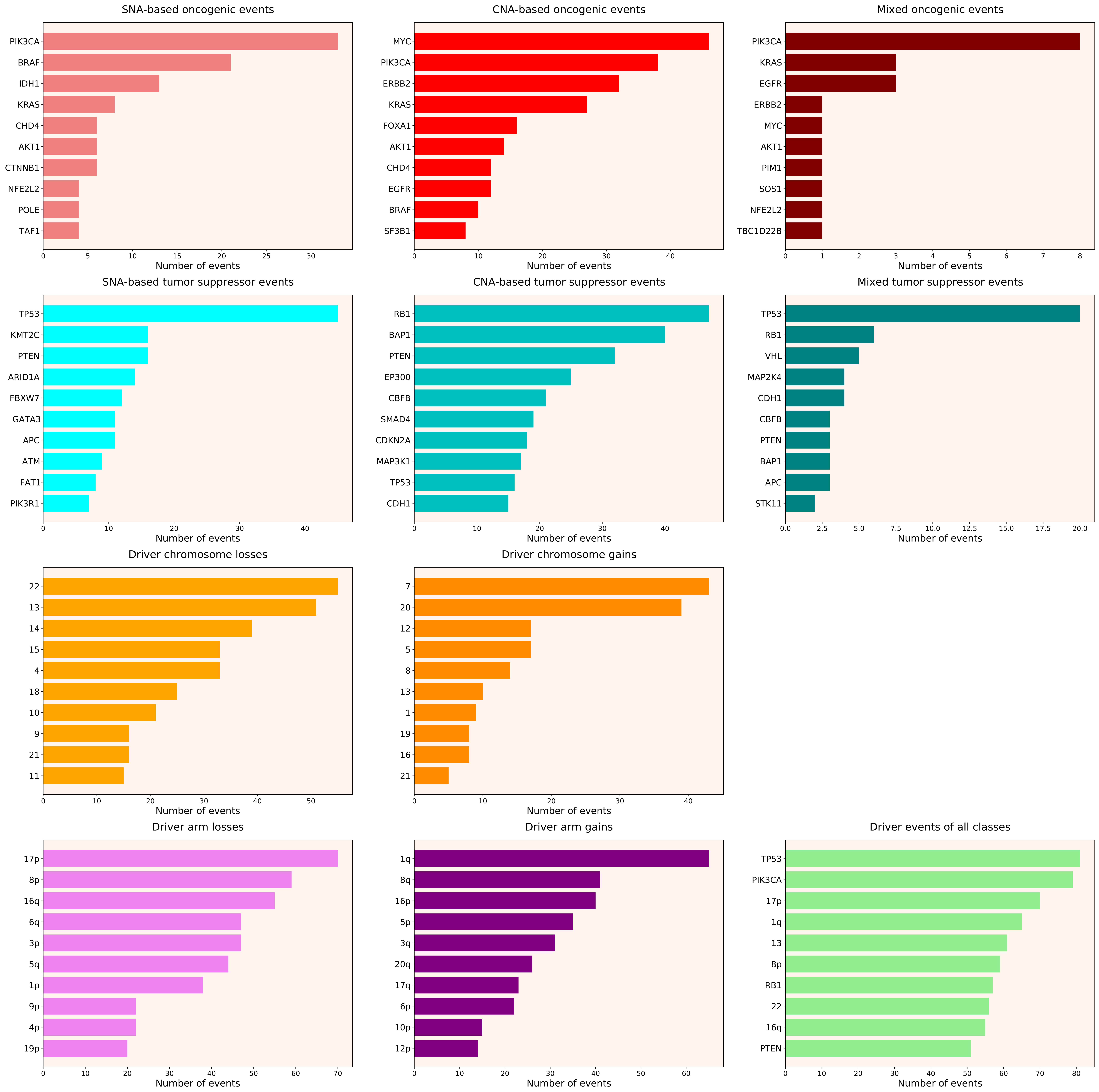
SNA-based oncogenic events
PIK3CA
BRAF
IDH1
KRAS
CHD4
AKT1
CTNNB1
NFE2L2
POLE
TAF1
0 5 10 15 20 25 30
Number of events
CNA-based oncogenic events
MYC
PIK3CA
ERBB2
KRAS
FOXA1
AKT1
CHD4
EGFR
BRAF
SF3B1
0 10 20 30 40
Number of events
Mixed oncogenic events
PIK3CA
KRAS
EGFR
ERBB2
MYC
AKT1
PIM1
SOS1
NFE2L2
TBC1D22B
0 1 2 3 4 5 6 7 8
Number of events
SNA-based tumor suppressor events
TP53
KMT2C
PTEN
ARID1A
FBXW7
GATA3
APC
ATM
FAT1
PIK3R1
0 10 20 30 40
Number of events
CNA-based tumor suppressor events
RB1
BAP1
PTEN
EP300
CBFB
SMAD4
CDKN2A
MAP3K1
TP53
CDH1
0 10 20 30 40
Number of events
Mixed tumor suppressor events
TP53
RB1
VHL
MAP2K4
CDH1
CBFB
PTEN
BAP1
APC
STK11
0.0 2.5 5.0 7.5 10.0 12.5 15.0 17.5 20.0
Number of events
Driver chromosome losses
22
13
14
15
4
18
10
9
21
11
0 10 20 30 40 50
Number of events
Driver chromosome gains
7
20
12
5
8
13
1
19
16
21
0 10 20 30 40
Number of events
Driver arm losses
17p
8p
16q
6q
3p
5q
1p
9p
4p
19p
0 10 20 30 40 50 60 70
Number of events
Driver arm gains
1q
8q
16p
5p
3q
20q
17q
6p
10p
12p
0 10 20 30 40 50 60
Number of events
Driver events of all classes
TP53
PIK3CA
17p
1q
13
8p
RB1
22
16q
PTEN
0 10 20 30 40 50 60 70 80
Number of events
